# Minimal Impact of Sensation‐Related Items on the Association Between Alexithymia and Self‐Report Measures of Interoception

**DOI:** 10.1002/pmh.70048

**Published:** 2025-11-12

**Authors:** Adam Ottley‐Porter, Kiera L. Adams, Rebecca Brewer, Jennifer Murphy

**Affiliations:** ^1^ Department of Psychology University of Surrey Guildford UK; ^2^ Department of Experimental Psychology University of Oxford Oxford UK; ^3^ Royal Holloway University of London Egham UK

**Keywords:** alexithymia, interoception, interoceptive accuracy, interoceptive attention, interoceptive sensibility, self‐reported interoception

## Abstract

Evidence suggests a relationship between alexithymia and self‐report measures of interoception. As measures of alexithymia often include items that may pick up on interoceptive difficulty, however, it is possible that previously reported associations are driven by a lack of independence of measurement. Here, we explored the effect of removing sensation‐related items from the Toronto Alexithymia Questionnaire (TAS‐20) on the association between the TAS‐20 and self‐report measures of interoceptive accuracy (Studies 1 and 2; *N* = 330 and *N* = 476, respectively) and attention (Study 2). In both studies, removal of sensation‐related items significantly reduced associations between the self‐report measures of interoception and alexithymia. This effect was specific to the removal of sensation‐related items (removing a random set of items did not result in a reduction in the size of the association). Importantly, relationships between alexithymia and self‐reported interoception remained after item removal. Although effects were modest, it is recommended that future studies exploring relationships with self‐report measures of interoception—particularly in relation to constructs where sensation‐related items may broadly feature—should implement sensitivity analyses or employ alternative instruments that exclude sensation‐related items, to ensure associations are not driven by a lack of independence of measurement.

## 1 Introduction

All theories of emotion ascribe a fundamental role to the processing of physical sensations in emotional experience (e.g., Damasio 1994; Gendron and Barrett 2009; James 1894; Schachter and Singer 1962). Consequently, there has been significant interest in examining the relationship between individual differences in emotion and interoception—the processing of internal bodily signals (Brewer et al. 2021). One area of focus has been the relationship between alexithymia and interoception. Alexithymia is a subclinical condition characterised by difficulties identifying and describing feelings, as well as an externally orientated thinking style (Apfel and Sifneos 1979; Nemiah et al. 1976). With respect to interoception, evidence consistently suggests individuals with higher rates of alexithymia—particularly cognitive dimensions (difficulties identifying and describing feelings)—self‐report difficulties perceiving interoceptive signals, suggesting poorer interoceptive accuracy (Brewer et al. 2016; Murphy et al. 2020; Trevisan et al. 2019; Gaggero et al. 2021). However, when assessed using behavioural tasks, results concerning the relationship between alexithymia and interoceptive accuracy are notably mixed (Borhani et al. 2017; Gaigg et al. 2018; Murphy, Brewer, et al. 2018; Murphy, Catmur, and Bird 2018; Trevisan et al. 2019).

Several explanations exist for the discrepancy between alexithymic individuals' self‐reported difficulties and their often‐typical performance on objective measures. Alexithymic individuals may lack insight into their ability (Scarpazza et al. 2022), behavioural tasks may be invalid (Desmedt et al. 2023; Murphy, Brewer, et al. 2018), or current measures (that often assess performance in one domain on a single occasion) may not be sensitive enough to capture the difficulties alexithymic individuals experience outside of the laboratory (Murphy 2023). Another possibility is that the associations between alexithymia and self‐report measures of interoception are inflated by a lack of measurement independence. Indeed, difficulties distinguishing bodily sensations and emotions are seen as a core feature of alexithymia (Fournier et al. 2019). As a result, measures of alexithymia such as the Toronto Alexithymia Questionnaire (TAS‐20; Bagby, Parker, and Taylor 1994; Bagby, Taylor, and Parker 1994) include items that are likely to pick up on interoceptive difficulty, such as ‘I have physical sensations that even doctors don't understand’ and ‘I am often puzzled by sensations in my body’. The same is true for other measures of alexithymia, such as the Bermond‐Vorst Alexithymia Questionnaire (BVAQ) and Perth Alexithymia Questionnaire (cognitive subscale), which also include ambiguous items that may pick up on interoceptive difficulty (Vorst and Bermond 2001; Preece et al. 2018). The extent to which measures include such ambiguous items may explain why the association between alexithymia and self‐reported interoception varies depending on the measure or subscales of alexithymia examined, with the affective and externally oriented thinking dimensions (e.g., BVAQ‐A; e.g., fantasising) typically showing weaker or absent relationships with self‐reported aspects of interoception (Gaggero et al. 2021). It remains unclear, however, whether the association between alexithymia and self‐reported interoception is fully explained by sensation‐related items that may capture interoceptive difficulties independently of emotional difficulties.

The aim of this series of studies was to examine the relationship between alexithymia and self‐reported interoceptive accuracy and attention, before and after removing items from the TAS‐20 that may tap into interoceptive difficulties (hereafter for brevity ‘sensation‐related items’). We focus on the TAS‐20 as this measure includes more items that explicitly reference bodily sensations. Study 1 examined the impact of sensation‐related item removal on the relationship between the TAS‐20 and the Interoceptive Accuracy Scale (IAS; Murphy et al. 2020) and compared this with random item removal. Study 2 replicated and extended these findings, also assessing relationships with the Interoceptive Attention Scale (IATS; Gabriele et al. 2020). As overlapping items may inflate associations (as shown in other areas, e.g., depression and sleep quality; Murphy, Wulff, et al. 2018), we predicted that removing sensation‐related items would reduce associations between the TAS‐20, IAS, and IATS. Given theoretical overlap between interoception and emotion (Brewer et al. 2021), however, we expected significant associations to remain.

## 2 Study 1

### 2.1 Methods

#### 2.1.1 Ethical Approval and Data Availability

Data for Study 1 were pooled from three projects, including one previously published dataset (Murphy et al. 2020). Ethical approval was granted by King's College London's ethics subcommittee (HR‐15/16‐2170). All participants provided informed consent, were fully debriefed after completing the study and were incentivised via a prize draw or course credits. Data for both studies are available at https://osf.io/hypjg/.

#### 2.1.2 Participants

Participants were recruited via social media, student participation systems, and preexisting databases of individuals who had expressed an interest in taking part in psychological research. After removal of incomplete records, data from 330 participants were available (*M*_Age_ = 28.50,^1^ *SD*_Age_ = 14.28, age range 18–91 years, 248 females, 81 males, 1 other). A total of 227 reported English as a first language, and 278 reported no psychiatric or neurodevelopmental diagnosis. Given that alexithymia co‐occurs with multiple psychiatric conditions (Brewer et al. 2021), and to ensure an adequate distribution of scores (Murphy, Brewer, et al. 2018), individuals reporting psychiatric conditions were retained.

#### 2.1.3 Materials

Alexithymia was assessed using the TAS‐20, a 20‐item measure assessing self‐reported difficulties identifying and describing one's own emotions, and externally oriented thinking (Bagby, Parker, and Taylor 1994; Bagby, Taylor, and Parker 1994). The TAS‐20 has excellent reliability and validity (Bagby, Parker, and Taylor 1994; Bagby, Taylor, and Parker 1994; Parker et al. 1993; present sample *α* = 0.88). Higher scores reflect greater alexithymia.

Self‐reported interoception was assessed using the IAS (Murphy et al. 2020), a 21‐item measure assessing self‐reported interoceptive accuracy. The IAS has good reliability and validity (Murphy et al. 2020; present sample *α* = .88), with higher scores reflecting better self‐perceived interoceptive accuracy.

#### 2.1.4 Procedure

Participants completed the IAS and TAS‐20 online via Qualtrics. Measures were presented in a randomised order, alongside other questionnaires.

#### 2.1.5 Data Analysis

Alexithymia was measured using the standard TAS‐20 total score (‘TAS‐Full’) and two modified scores with specific items removed. For the ‘TAS‐NS’ (TAS with no sensory items), we removed items 3, 7 and 13 (all from the difficulties identifying feelings subscale), which likely reflect interoceptive difficulties (e.g., ‘I am often puzzled by sensations in my body’). Items for removal were selected through discussion amongst all authors in consultation with two experts in the field of alexithymia. Removal of items 3 and 7 was supported by research using factor analysis, suggesting overlap between items 3 and 7 and questionnaire items that may tap interoceptive difficulties (e.g., anxiety questionnaires; Fournier et al. 2019). As factor analyses may be influenced by the content of the other questionnaires included, however, and as all authors considered item 13 (‘I don't know what's going on inside me’) to have a sensory component, this item was also removed. For the ‘TAS‐NPS’ (TAS with no *potentially* sensory items), we further excluded items 2, 4 and 9 (all from the difficulties identifying and describing feelings subscales), which may also tap interoceptive difficulty (e.g., ‘I have feelings that I can't quite identify’). To ensure that differences in associations were specific to the removal of sensation‐related items rather than changes to psychometric properties, we generated 10 additional scores by randomly removing 3–6 items (avoiding the six sensation‐related items above) using an online random number generator (https://www.random.org/).

#### 2.1.6 Results

Descriptive statistics and Pearson correlations examining associations between the IAS and TAS‐20 (before and after item removal) are presented in Table 1. Significant correlations were observed between all variables, with significant negative associations between the TAS‐20 and IAS even after removing sensation‐related items.

**TABLE 1:** Descriptive statistics and correlations between key variables.

Variable	Mean	SD	Range	1	2	3	4
1. IAS	81.93	10.399	44–105	1	−0.444*	−0.425*	−0.388*
2. TAS‐FULL	48.70	12.653	20–80		1	0.985*	0.949*
3. TAS‐NS	41.90	10.770	17–70			1	0.977*
4. TAS‐NPS	33.56	8.392	14–56				1

*Note:* Significant associations at *p* < 0.05 are denoted by *.

Abbreviations: IAS, Interoceptive Accuracy Scale; TAS‐20, full TAS‐20 as typically calculated; TAS‐NPS, TAS‐20 after the removal of items that could possibly pick up on interoceptive difficulty; TAS‐NS, TAS‐20 after the removal of items likely to pick up on interoceptive difficulty (see text for details).

To examine whether the removal of sensation‐related items had a significant effect on the association between TAS‐20 and IAS scores, correlations were compared using Steiger's (1980) *Z*‐test implemented using an online calculator (Lee and Preacher 2013). Predicting lower associations post‐item removal, we employed one‐tailed tests. Results revealed a significantly stronger association between the IAS and TAS‐Full than between the IAS and TAS‐NS (*Z* = −2.208, *p* = 0.014) and between the IAS and TAS‐NPS (*Z* = −3.51, *p* < 0.001).

To explore whether this effect was specific to the removal of sensation‐related items, we examined the impact of random removal of items from the TAS‐20 (see Tables S1 and S2). The correlation between these total scores after random item removal and the IAS was then compared with the correlation between the TAS‐Full and IAS. As before, one‐tailed tests were used to determine whether random item removal reduced associations between alexithymia and IAS scores (as found with the TAS‐NS and TAS‐NPS). Where scores were inflated (likely because more sensation‐related items were retained), these were noted as nonsignificant given directional predictions. As detailed in Supplement Table S1, when removing three items (as in TAS‐NS), only 2/10 comparisons were significant, although in the opposite direction to hypothesised, thereby not indicating a reduction in the association following random item removal. When removing six items (as in TAS‐NPS), only 2/10 comparisons were significant. One of these indicated that the association was inflated following item removal, but one (removing items 5, 14, 8, 10, 1, 16) led to a significant reduction in the association with the IAS.

#### 2.1.7 Interim Discussion

In Study 1, we observed that removing sensation‐related items from the TAS‐20 significantly reduced associations between alexithymia and the IAS. For conservative sensation‐related item selection, this reduction appeared specific to the removal of sensation‐related items. For more liberal removal, however, there was some indication that this may not be specific. These results indicate that the association between alexithymia and self‐reported interoceptive accuracy is inflated by items on the TAS‐20 that likely pick up on interoceptive difficulty. Notably, although significant, this inflation was small (representing an overall increase in the effect size of *r* = 0.06), and importantly, the association remained significant after the removal of items that may tap interoceptive difficulty. Evidence indicates, however, that self‐reported interoception dissociates across dimensions; beliefs about one's accuracy do not correspond with those regarding one's interoceptive attention (Gabriele et al. 2020; Murphy et al. 2020). Thus, it remains unclear whether these effects are restricted to measures of self‐reported interoceptive accuracy. Study 2 explored this question and provided a replication of Study 1.

## 3 Study 2

### 3.1 Methods

#### 3.1.1 Ethical Approval and Data Availability

Study 2 received ethical approval from the Medical Sciences Interdivisional Research Ethics Committee (IDREC; R79981/RE005) at The University of Oxford. All participants provided informed consent and were fully debriefed. Participation was incentivised via a prize draw or course credits.

#### 3.1.2 Participants

Data from Study 2 were taken from a larger study examining the overlap between autism and mental health conditions. Participants were recruited via social media, adverts via charity partners, student participation systems and preexisting databases of individuals who had indicated an interest in taking part in psychological research. After the removal of incomplete records and those where reliability could not be established,^2^ data were available from 476 participants (*M*_Age_ = 36.07, *SD*_Age_ = 14.80, age range 18–82 years, 337 females, 92 males, 47 other). Eighty‐seven participants reported no psychiatric or neurodevelopmental diagnosis, as expected given targeted recruitment strategies.

#### 3.1.3 Materials

For brevity, only the measures relevant to this study are discussed. In addition to the aforementioned questionnaires (see Study 1), in Study 2, we also included the Interoceptive Attention Scale (IATS; Gabriele et al. 2020). This 21‐item measure has good reliability and validity (Gabriele et al. 2020) and assesses the degree to which interoceptive signals are the object of one's attention, with higher scores reflecting greater self‐reported interoceptive attention. This questionnaire exactly matches the IAS sensations while assessing a unique dimension of interoceptive processing dissociable from self‐reported interoceptive accuracy (Gabriele et al. 2020; Murphy et al. 2020).

#### 3.1.4 Procedure

Participants completed the IAS (present sample *α* = 0.90), IATS (present sample *α* = 0.89) and TAS‐20 (present sample *α* = 0.99), online via Qualtrics as part of a larger set of measures. Unlike Study 1, the TAS‐20's positively worded items preceded the negatively worded ones due to researcher error.

#### 3.1.5 Data Analysis

TAS‐Full, TAS‐NS and TAS‐NPS scores were calculated as per Study 1. Ten additional scores were created by randomly removing the same TAS‐20 items as in Study 1.

#### 3.1.6 Results

Descriptive statistics and Pearson correlations examining associations between the IAS, IATS and TAS‐20 (before and after the removal of items) are presented in Table 2. No association was observed between the IAS and IATS. Replicating Study 1, significant negative associations were observed between the TAS‐20 and IAS even after removing sensation‐related items. In contrast, significant positive correlations were observed between the TAS‐20 and IATS even after removing sensation‐related items.

**TABLE 2:** Descriptive statistics and correlations between all key variables.

Variable	Mean	SD	Range	1	2	3	4	5
1. IAS	71.99	14.023	21–105	1	−0.066	−0.487**	−0.455**	−0.418**
2. IATS	50.49	14.362	21–100		1	0.208**	0.149**	0.130**
3. TAS‐FULL	59.52	13.880	20–89			1	0.983**	0.960**
4. TAS‐NS	49.64	11.758	17–76				1	0.985**
5. TAS‐NPS	38.88	9.211	14–61					1

*Notes:* Significant associations at *p* < 0.05 are denoted by **.

Abbreviations: IAS, Interoceptive Accuracy Scale; IAS, Interoceptive Attention Scale; TAS‐20, full TAS‐20 as typically calculated. TAS‐NPS, TAS‐20 after the removal of items that could possibly pick up on interoceptive difficulty; TAS‐NS, TAS‐20 after the removal of items likely to pick up on interoceptive difficulty (see text for details).

As in Study 1, correlations were compared using Steiger's (1980) *Z*‐test implemented using an online calculator (Lee and Preacher 2013). Results revealed a significantly stronger association between the IAS and TAS‐Full than between the IAS and TAS‐NS (*Z* = −4.303, *p* ≤ 0.001) and between the IAS and TAS‐NPS (*Z* = −6.017, *p* < 0.001). For the IATS, results revealed a significantly stronger association between the IATS and TAS‐Full than between the IATS and TAS‐NS (*Z* = 7.09, *p* < 0.001) and between the IATS and TAS‐NPS (*Z* = 6.101, *p* < 0.001).

As in Study 1, we explored whether this effect was specific to the removal of sensation‐related items (see Tables S3–S6). For both the IAS and IATS, where significant differences were observed all indicated that the relationship between alexithymia and IAS/IATS scores was inflated following item removal and was thus treated as nonsignificant given directional predictions. Notably, the significant result for one comparison in Study 1 did not replicate in Study 2. Considering the number of comparisons tested and the lack of replication, the *p* = 0.025 finding in Study 1 is best interpreted as a probable false positive and was therefore not considered further.

#### 3.1.7 Interim Discussion

Replicating and extending the results of Study 1, we observed that removing sensation‐related items from the TAS‐20 significantly reduced associations between alexithymia and both the IAS and IATS. Importantly, this was specific to the removal of items likely to pick up on interoceptive difficulty. As expected, the IAS and IATS were uncorrelated and showed differential relationships with alexithymia (Gabriele et al. 2020; Murphy et al. 2020).

## 4 General Discussion

The aim of this pair of studies was to examine whether the relationship between alexithymia and self‐report measures of interoception may be inflated by items on alexithymia questionnaires that may tap into interoceptive difficulty. In Study 1, removing such items reduced the association between the TAS‐20 and IAS. This was replicated in Study 2, where we also showed similar effects for the IATS. Follow‐up analyses demonstrated that this result was broadly specific to the removal of sensation‐related items, items that feature on alexithymia subscales such as difficulties identifying and describing feelings, but not externally oriented thinking. Importantly, whilst item removal significantly reduced associations, relationships remained after item removal, suggesting that relationships are not solely driven by the presence of items on alexithymia questionnaires that relate to interoceptive difficulty.

These results have important broader implications for research employing self‐report interoception measures, particularly for studies of anxiety and depression where questionnaires often include items that may tap interoceptive processes (see Clemente et al. 2024). Indeed, common measures such as the GAQ‐7 (Spitzer et al. 2006) and PHQ‐9 (Kroenke et al. 2001) include measures that may tap interoceptive difficulty (e.g., difficulties with sleep, energy levels, appetite or restlessness). Although we observed that item removal only resulted in a modest reduction in the association between alexithymia and self‐reported interoception, effects may be larger for questionnaires where many items tap into interoceptive difficulty. For studies seeking to explore relationships between self‐report measures of interoception and other questionnaires where such items may feature (e.g., depression or anxiety measures), it is recommended that studies select questionnaires that do not contain, or contain few, items relating to interoceptive difficulty (e.g., Hospital Anxiety and Depression Scale; Perth Alexithymia Questionnaire; Preece et al. 2018; Preece et al. 2023; Zigmond and Snaith 1983), or at a minimum conduct sensitivity analyses removing relevant items that may drive associations.

These data also speak to the relationship between self‐report measures of interoception and their relationship with alexithymia. Consistent with prior literature, we observed no association between the IAS and IATS, indicating a dissociation between beliefs about accuracy and attention (Gabriele et al. 2020; Murphy et al. 2020). Differential relationships with alexithymia underscore the need to assess these facets separately; as expected, alexithymia was associated with lower self‐reported interoceptive accuracy and greater self‐reported attention to bodily sensations (Brewer et al. 2016, 2021; Murphy et al. 2020; Trevisan et al. 2019; Van Bael et al. 2024). Given that previous findings suggest heightened attention may be driven by co‐occurring anxiety (Murphy et al. 2020), however, this effect may not be solely attributable to alexithymia. Importantly, both effects persisted following item removal, indicating that alexithymia is linked to both reduced interoceptive accuracy and elevated attention, consistent with established theories of emotion (e.g., Damasio 1994; Gendron and Barrett 2009; James 1894; Schachter and Singer 1962).

In conclusion, our findings indicate that sensation‐related items on alexithymia questionnaires may inflate associations with self‐report measures of interoception. Although effects were modest, future studies exploring relationships with self‐report measures of interoception—particularly in relation to constructs such as anxiety or depression—should implement sensitivity analyses or employ alternative instruments that exclude sensation‐related items. Such recommendations are essential to ensure that a lack of independence of measurement does not drive associations.

## Author Contributions

JM conceived the idea, completed analyses, drafted the manuscript and collected data for Study 1. AOP assisted with analyses and critical edits to early drafts of the manuscript. JM, AOP and RB assessed items for removal. KLA collected data for Study 2. All authors provided comments on the final manuscript draft.

## Ethics Statement

Ethical approval was provided by the local ethics subcommittees. All participants provided informed written consent and were debriefed on completion.

## Conflicts of Interest

JM has completed paid consultancy work for Healios for work on interoception.

## Statements and Declarations

JM has completed paid consultancy work for Healios for work on interoception. JM is supported by a New Investigator Research Grant from the Medical Research Council (MR/X010295/1). KA is supported by a University of Oxford Medical Sciences Graduate School Studentship.

## Supporting information


**Table S1:** Results after the removal of three random items (excluding sensation‐related items) for Study 1.
**Table S2:** Results after the removal of six random items (excluding sensation‐related items) for Study 1.
**Table S3:** Results after the removal of three random items (excluding sensation‐related items) for Study 2.
**Table S4:** Results after the removal of six random items (excluding sensation‐related items) for Study 2.
**Table S5:** Results after the removal of three random items (excluding sensation‐related items) for Study 2.
**Table S6:** Results after the removal of six random items (excluding sensation‐related items) for Study 2.

## Data Availability

Data are openly available via the links provided in the manuscript.

## Endnotes

^1^
Age data were missing for five participants.

^2^
Data were excluded if participants failed reCAPTCHA checks, if gibberish had been entered into any of the open text options, or if response times were under 10 min.
