# Supplementary material for: Minimal Impact of Sensation‐Related Items on the Association Between Alexithymia and Self‐Report Measures of Interoception
Source: Personal Ment Health. 2025 Nov 12;19(4):e70048. doi: 10.1002/pmh.70048 (PMC12606692; doi:10.1002/pmh.70048)
Supplement: Supplementary file 1 — Table S1: Results after the removal of three random items (excluding sensation‐related items) for Study 1. Table S2: Results after the removal of six random items (excluding sensation‐related items) for Study 1. Table S3: Results after the removal of three random items (excluding sensation‐related items) for Study 2. Table S4: Results after the removal of six random items (excluding sensation‐related items) for Study 2. Table S5: Results after the removal of three random items (excluding sensation‐related items) for Study 2. Table S6: Results after the removal of six random items (excluding sensation‐related items) for Study 2. [file PMH-19-0-s001.docx]

**SUPPLEMENT: Minimal Impact of Sensation-Related Items on the Association Between Alexithymia and Self-report Measures of Interoception**

Adam Ottley-Porter^1^, Kiera L. Adams^2^, Rebecca Brewer^3^ & Jennifer Murphy^1,*^

^1^Department of Psychology, University of Surrey, Guildford, UK

^2^Department of Experimental Psychology, University of Oxford

^3^Royal Holloway University of London, Egham, UK

*Corresponding Author

Jennifer Murphy

Department of Psychology

University of Surrey

Guildford, UK

[Jennifer.murphy@surrey.ac.uk](mailto:Jennifer.murphy@surrey.ac.uk)

**Table S1.** Results after the removal of three random items (excluding sensation-related items) for Study One.

| **Variable** | **Items** | **%DIF** | **%DDF** | **%EOT** | **Mean** | **SD** | **Range** | **R-IAS** | **R-TASFull** | **Z** | **P** |
| --- | --- | --- | --- | --- | --- | --- | --- | --- | --- | --- | --- |
| 1 | 1, 20, 11 | 33.3% | 33.3% | 33.3% | 41.16 | 10.703 | 17-68 | -.445^*^ | .991^*^ | .151 | .440 |
| 2 | 14, 18, 8 | 33.3% | 0% | 66.7% | 42.24 | 11.438 | 17-71 | -.433^*^ | .990^*^ | -1.567 | .059 |
| 3 | 1, 15, 18 | 33.3% | 0% | 66.7% | 41.42 | 11.076 | 17-67 | -.440^*^ | .993^*^ | -.682 | .248 |
| 4 | 18, 16, 8 | 0% | 0% | 100% | 41.89 | 11.802 | 17-71 | -.445^*^ | .989^*^ | .136 | .446 |
| 5 | 12, 10, 20 | 0% | 33.3% | 66.7% | 41.85 | 11.223 | 17-72 | -.456^*^ | .988^*^ | 1.571 | .058 |
| 6 | 6, 15, 17 | 33.3% | 33.3% | 33.3% | 40.56 | 10.483 | 17-66 | -.464^*^ | .990^*^ | 2.88 | .002 (NS)^ |
| 7 | 10, 17, 20 | 0% | 33.3% | 66.7% | 41.38 | 11.149 | 17-69 | -.467^*^ | .987^*^ | 2.908 | .002 (NS)^ |
| 8 | 11, 12, 5 | 0% | 66.7% | 33.3% | 41.57 | 10.819 | 17-70 | -.449^*^ | .991^*^ | .754 | .226 |
| 9 | 10, 14, 15 | 33.3% | 0% | 66.7% | 41.68 | 11.059 | 17-68 | -.443^*^ | .989^*^ | -.136 | .446 |
| 10 | 5, 10, 8 | 0% | 0% | 100% | 42.48 | 11.853 | 17-73 | -.447^*^ | .990^*^ | .429 | .334 |

*Note.* R-IAS = correlation between the TAS-20 scores after the removal of items listed in column 1 and the IAS. R-TASFull = correlation between the TAS-20 scores after the removal of items listed in column and the TAS-Full. DIF = Difficulties identifying feelings subfactor. DDF = Difficulties describing feelings subfactor. EOT = Externally Orientated Thinking. *denotes significance at p<.05. ^given directional predictions, only where a significant reduction in the size of the correlation was observed are noted as significant.

**Table S2.** Results after the removal of six random items (excluding sensation-related items) for Study One.

| **Variable** | **Items** | **%DIF** | **%DDF** | **%EOT** | **Mean** | **SD** | **Range** | **R-IAS** | **R-TASFull** | **Z** | **P** |
| --- | --- | --- | --- | --- | --- | --- | --- | --- | --- | --- | --- |
| 1 | 8, 11, 20, 17, 1, 16 | 16.7% | 33.3% | 50.0% | 33.32 | 9.206 | 14-59 | -.465^*^ | .975^*^ | 1.912 | .028 (NS)^ |
| 2 | 12, 14, 16, 19, 8, 5 | 16.7% | 16.7% | 66.7% | 34.72 | 9.896 | 14-62 | -.426^*^ | .975^*^ | -1.62 | .053 |
| 3 | 5, 14, 8, 10, 1, 16 | 33.3% | 0% | 66.7% | 34.92 | 9.992 | 14-61 | -.425^*^ | .981^*^ | -1.961 | **.025*** |
| 4 | 15, 16, 6, 11, 17, 14 | 33.3% | 33.3% | 33.3% | 33.01 | 8.584 | 14-54 | -.459^*^ | .974^*^ | 1.336 | .091 |
| 5 | 14, 1, 17, 12, 15, 10 | 33.3% | 33.3% | 33.3% | 33.64 | 8.628 | 14-55 | -.448^*^ | .971^*^ | .336 | .368 |
| 6 | 20, 6, 1, 10, 19, 16 | 33.3% | 0% | 66.7% | 34.47 | 9.647 | 14-61 | -.434^*^ | .980^*^ | -1.008 | .157 |
| 7 | 18, 14, 17, 12, 1, 10 | 33.3% | 33.3% | 33.3% | 34.46 | 8.912 | 14-55 | -.437^*^ | .978^*^ | -.673 | .250 |
| 8 | 1, 15, 18, 5, 12, 6 | 33.3% | 16.7% | 50.0% | 34.44 | 9.339 | 14-58 | -.437^*^ | .985^*^ | -.815 | .208 |
| 9 | 17, 16, 14, 8, 10, 19 | 16.7% | 16.7% | 66.7% | 34.38 | 9.631 | 14-58 | -.445^*^ | .974^*^ | .089 | .465 |
| 10 | 16, 5, 1, 18, 20,11 | 16.7% | 16.7% | 66.7% | 34.48 | 9.963 | 14-60 | -.442^*^ | .980^*^ | -.202 | .420 |

*Note.* R-IAS = correlation between the TAS-20 scores after the removal of items listed in column 1 and the IAS. R-TASFull = correlation between the TAS-20 scores after the removal of items listed in column and the TAS-Full. DIF = Difficulties identifying feelings subfactor. DDF = Difficulties describing feelings subfactor. EOT = Externally Orientated Thinking. *denotes significance at p<.05. ^given directional predictions, only where a significant reduction in the size of the correlation was observed are noted as significant.

**Table S3.** Results after the removal of three random items (excluding sensation-related items) for Study Two.

| **Variables** | **Items** | **%DIF** | **%DDF** | **%EOT** | **Mean** | **SD** | **Range** | **R-IAS** | **R-TASFull** | **Z** | **P** |
| --- | --- | --- | --- | --- | --- | --- | --- | --- | --- | --- | --- |
| 1 | 1, 20, 11 | 33.3% | 33.3% | 33.3% | 50.03 | 11.472 | 17-75 | -.486^*^ | .993^*^ | -.211 | .417 |
| 2 | 14, 18, 8 | 33.3% | 0% | 66.7% | 52.41 | 12.525 | 17-77 | -.485^*^ | .991^*^ | -.371 | .355 |
| 3 | 1, 15, 18 | 33.3% | 0% | 66.7% | 50.82 | 11.941 | 17-77 | -.489^*^ | .993^*^ | .421 | .337 |
| 4 | 18, 16, 8 | 0% | 0% | 100% | 52.48 | 13.048 | 17-79 | -.492^*^ | .989^*^ | .842 | .200 |
| 5 | 12, 10, 20 | 0% | 33.3% | 66.7% | 51.73 | 11.974 | 17-78 | -.500^*^ | .992^*^ | 2.576 | .005 (NS)^ |
| 6 | 6, 15, 17 | 33.3% | 33.3% | 33.3% | 49.51 | 11.473 | 17-74 | -.501^*^ | .991^*^ | 2.617 | .004 (NS)^ |
| 7 | 10, 17, 20 | 0% | 33.3% | 66.7% | 51.03 | 12.103 | 17-78 | -.505^*^ | .991^*^ | 3.373 | .001 (NS)^ |
| 8 | 11, 12, 5 | 0% | 66.7% | 33.3% | 51.34 | 11.977 | 17-76 | -.493^*^ | .993^*^ | 1.266 | .103 |
| 9 | 10, 14, 15 | 33.3% | 0% | 66.7% | 51.21 | 11.925 | 17-78 | -.496^*^ | .991^*^ | 1.678 | .047 (NS)^ |
| 10 | 5, 10, 8 | 0% | 0% | 100% | 53.36 | 13.164 | 17-80 | -.492^*^ | .991^*^ | .93 | .176 |

*Note. R-IAS = correlation between the TAS-20 scores after the removal of items listed in column 1 and the IAS. R-TASFull = correlation between the TAS-20 scores after the removal of items listed in column and the TAS-Full. DIF = Difficulties identifying feelings subfactor. DDF = Difficulties describing feelings subfactor. EOT = Externally Orientated Thinking. *denotes significance at p<.05. ^given directional predictions, only where a significant reduction in the size of the correlation was observed are noted as significant.*

**Table S4.** Results after the removal of six random items (excluding sensation-related items) for Study Two.

| **Variable** | **Items** | **%DIF** | **%DDF** | **%EOT** | **Mean** | **SD** | **Range** | **R-IAS** | **R-TASFull** | **Z** | **P** |
| --- | --- | --- | --- | --- | --- | --- | --- | --- | --- | --- | --- |
| 1 | 8, 11, 20, 17, 1, 16 | 16.7% | 33.3% | 50.0% | 41.51 | 10.236 | 14-65 | -.507^*^ | .977^*^ | 2.345 | .010 (NS)^ |
| 2 | 12, 14, 16, 19, 8, 5 | 16.7% | 16.7% | 66.7% | 44.59 | 11.184 | 14-66 | -.493^*^ | .976^*^ | .685 | .247 |
| 3 | 5, 14, 8, 10, 1, 16 | 33.3% | 0% | 66.7% | 44.11 | 11.191 | 14-67 | -.481^*^ | .981^*^ | -.766 | .222 |
| 4 | 15, 16, 6, 11, 17, 14 | 33.3% | 33.3% | 33.3% | 40.28 | 9.446 | 14-61 | -.502^*^ | .976^*^ | 1.718 | .043 (NS)^ |
| 5 | 14, 1, 17, 12, 15, 10 | 33.3% | 33.3% | 33.3% | 41.21 | 9.398 | 14-63 | -.506^*^ | .978^*^ | 2.277 | .011 (NS)^ |
| 6 | 20, 6, 1, 10, 19, 16 | 33.3% | 0% | 66.7% | 42.64 | 10.206 | 14-64 | -.475^*^ | .982^*^ | -1.572 | .058 |
| 7 | 18, 14, 17, 12, 1, 10 | 33.3% | 33.3% | 33.3% | 42.15 | 9.718 | 14-66 | -.488^*^ | .982^*^ | .131 | .448 |
| 8 | 1, 15, 18, 5, 12, 6 | 33.3% | 16.7% | 50.0% | 42.77 | 10.034 | 14-64 | -.483^*^ | .986^*^ | -.595 | .276 |
| 9 | 17, 16, 14, 8, 10, 19 | 16.7% | 16.7% | 66.7% | 43.42 | 10.884 | 14-65 | -.500^*^ | .974^*^ | 1.429 | .076 |
| 10 | 16, 5, 1, 18, 20,11 | 16.7% | 16.7% | 66.7% | 43.20 | 10.791 | 14-68 | -.487^*^ | .984^*^ | 0 | .500 |

*Note.* R-IAS = correlation between the TAS-20 scores after the removal of items listed in column 1 and the IAS. R-TASFull = correlation between the TAS-20 scores after the removal of items listed in column and the TAS-Full. DIF = Difficulties identifying feelings subfactor. DDF = Difficulties describing feelings subfactor. EOT = Externally Orientated Thinking. *denotes significance at p<.05. ^given directional predictions, only where a significant reduction in the size of the correlation was observed are noted as significant.

**Table S5.** Results after the removal of three random items (excluding sensation-related items) for Study Two.

| **Variable** | **Items** | **%DIF** | **%DDF** | **%EOT** | **Mean** | **SD** | **Range** | **R-IATS** | **R-TASFull** | **Z** | **P** |
| --- | --- | --- | --- | --- | --- | --- | --- | --- | --- | --- | --- |
| 1 | 1, 20, 11 | 33.3% | 33.3% | 33.3% | 50.03 | 11.472 | 17-75 | .216^*^ | .993^*^ | -1.505 | .066 |
| 2 | 14, 18, 8 | 33.3% | 0% | 66.7% | 52.41 | 12.525 | 17-77 | .201^*^ | .991^*^ | 1.159 | .123 |
| 3 | 1, 15, 18 | 33.3% | 0% | 66.7% | 50.82 | 11.941 | 17-77 | .222^*^ | .993^*^ | -2.636 | .004 (NS)^ |
| 4 | 18, 16, 8 | 0% | 0% | 100% | 52.48 | 13.048 | 17-79 | .215^*^ | .989^*^ | -1.05 | .147 |
| 5 | 12, 10, 20 | 0% | 33.3% | 66.7% | 51.73 | 11.974 | 17-78 | .225 | .992^*^ | -2.996 | .001 (NS)^ |
| 6 | 6, 15, 17 | 33.3% | 33.3% | 33.3% | 49.51 | 11.473 | 17-74 | .220^*^ | .991^*^ | -1.992 | .023 (NS)^ |
| 7 | 10, 17, 20 | 0% | 33.3% | 66.7% | 51.03 | 12.103 | 17-78 | .229^*^ | .991^*^ | -3.491 | .001 (NS)^ |
| 8 | 11, 12, 5 | 0% | 66.7% | 33.3% | 51.34 | 11.977 | 17-76 | .210^*^ | .993^*^ | -.376 | .353 |
| 9 | 10, 14, 15 | 33.3% | 0% | 66.7% | 51.21 | 11.925 | 17-78 | .225^*^ | .991^*^ | -2.824 | .002 (NS)^ |
| 10 | 5, 10, 8 | 0% | 0% | 100% | 53.36 | 13.164 | 17-80 | .222^*^ | .991^*^ | -2.325 | .010 (NS)^ |

*Note.* R-IATS = correlation between the TAS-20 scores after the removal of items listed in column 1 and the IATS. R-TASFull = correlation between the TAS-20 scores after the removal of items listed in column and the TAS-Full. DIF = Difficulties identifying feelings subfactor. DDF = Difficulties describing feelings subfactor. EOT = Externally Orientated Thinking. *denotes significance at p<.05. ^given directional predictions, only where a significant reduction in the size of the correlation was observed are noted as significant.

**Table S6.** Results after the removal of six random items (excluding sensation-related items) for Study Two.

| **Variable** | **Items** | **%DIF** | **%DDF** | **%EOT** | **Mean** | **SD** | **Range** | **R-IATS** | **R-TASFull** | **Z** | **P** |
| --- | --- | --- | --- | --- | --- | --- | --- | --- | --- | --- | --- |
| 1 | 8, 11, 20, 17, 1, 16 | 16.7% | 33.3% | 50.0% | 41.51 | 10.236 | 14-65 | .228^*^ | .977^*^ | -2.079 | .019 (NS)^ |
| 2 | 12, 14, 16, 19, 8, 5 | 16.7% | 16.7% | 66.7% | 44.59 | 11.184 | 14-66 | .223^*^ | .976^*^ | -1.526 | .064 |
| 3 | 5, 14, 8, 10, 1, 16 | 33.3% | 0% | 66.7% | 44.11 | 11.191 | 14-67 | .220^*^ | .981^*^ | -1.371 | .085 |
| 4 | 15, 16, 6, 11, 17, 14 | 33.3% | 33.3% | 33.3% | 40.28 | 9.446 | 14-61 | .219^*^ | .976^*^ | -1.118 | .131 |
| 5 | 14, 1, 17, 12, 15, 10 | 33.3% | 33.3% | 33.3% | 41.21 | 9.398 | 14-63 | .231^*^ | .978^*^ | -2.446 | .007 (NS)^ |
| 6 | 20, 6, 1, 10, 19, 16 | 33.3% | 0% | 66.7% | 42.64 | 10.206 | 14-64 | .250^*^ | .982^*^ | -4.955 | <.001(NS)^ |
| 7 | 18, 14, 17, 12, 1, 10 | 33.3% | 33.3% | 33.3% | 42.15 | 9.718 | 14-66 | .215^*^ | .982^*^ | -.821 | .206 |
| 8 | 1, 15, 18, 5, 12, 6 | 33.3% | 16.7% | 50.0% | 42.77 | 10.034 | 14-64 | .218^*^ | .986^*^ | -1.331 | .092 |
| 9 | 17, 16, 14, 8, 10, 19 | 16.7% | 16.7% | 66.7% | 43.42 | 10.884 | 14-65 | .241^*^ | .974^*^ | -3.233 | .001 (NS)^ |
| 10 | 16, 5, 1, 18, 20,11 | 16.7% | 16.7% | 66.7% | 43.20 | 10.791 | 14-68 | .223^*^ | .984^*^ | -1.868 | .031 (NS)^ |

*Note.* R-IATS = correlation between the TAS-20 scores after the removal of items listed in column 1 and the IATS. R-TASFull = correlation between the TAS-20 scores after the removal of items listed in column and the TAS-Full. DIF = Difficulties identifying feelings subfactor. DDF = Difficulties describing feelings subfactor. EOT = Externally Orientated Thinking. *denotes significance at p<.05. ^given directional predictions, only where a significant reduction in the size of the correlation was observed are noted as significant.
